# A Few Words Anent Wolf-Teeth

**Published:** 1898-02

**Authors:** L. A. Merillat

**Affiliations:** Professor of Dental Surgery, M’killip Veterinary College, Chicago


### THE JOURNAL OF COMPARATIVE MEDICINE AND VETERINARY ARCHIVES.

VOL. XIX. *FEBRUARY, 1898.* No. 2.

### A FEW WORDS ANENT WOLF-TEETH.

By Dr. L. A. Merillat,
PROFESSOR OF DENTAL SURGERY, M'KILLIP VETERINARY COLLEGE, CHICAGO.

*Synonyms:* Rudimentary premolars, anterior premolars, undeveloped premolars, retrogressive premolars, supernumerary teeth, supplementary teeth, remnant-teeth, eye-teeth.

*Definition.* Wolf-teeth, so called because they bear some resemblance to the canines of the dog or wolf species, are retrogressive dental structures undergoing degeneration in the modern domestic horse, and representing the anterior premolars of extinct species.

*Anatomy.* They are located in the interdental space of the upper jaw, rarely the lower, usually related to the anterior border of the first molar, but may occur in any part of the interdental space behind the canine. The common location of those not contiguous to the molar is from one-half to four centimetres from it.

*Size.* The variations in their size, shape, and structure, due, no doubt, to the varied integrity of their surroundings, their poor individuality, and their tendency to degenerate, exceed those of all other normal structures. A majority of the observable ones vary from four to thirty millimetres in length and from two to eight millimetres in diameter. Others, favored with influences conducive to dental development, attain the size of a canine, and in rare cases assume the characteristics of herbivorous molars, while still others never reach the period of eruption, but remain invisible to casual inspection.

*Shape.* In shape they vary somewhat less than in size, resembling a human canine-tooth in many respects. They present a well-marked neck, fang, and crown, and in this respect seem to represent teeth of temporary dentition. Their crowns, divided from the fang by a well-restricted neck, are varied in shape, but always irregular, and if closely related to the molar may present a flat posterior surface. The fang constitutes the major portion of the tooth. It tapers to a blunt, rounded extremity, is irregular, often curved, and is always covered with rough incrustations of cement. It has no foramen at its apex, as the pulp-cavity is obliterated at an early stage of development, if it exists at all.

*Structure.* The better-developed ones are composed of the fundamental dental substances, normally arranged. The enamel-organ forms a cap for the crown identical with the enamel-cap of the human molar; but, unlike the latter, it soon wears so as to expose the dentine—an herbivorous characteristic. It does not, however, extend beyond the neck, as in the typical herbivorous molar.

The dentine constitutes the bulk of the tooth, seldom interrupted by an infundibulum or a pulp-cavity.

The cement forms a thin layer over the neck and fang. It serves to increase the solidity of its implantation, and is the only medium through which the tooth obtains nutriment.

In fine, their structure resembles the simplest form of mammalian teeth—*i. e.*, a body of dentine capped with enamel and incrusted over the remaining portion with cement.

The very rudimentary ones show no regular arrangement of the fundamental substances, but consist of a homogeneous mass of dentine, dense on the exterior, and becoming less so toward a central white cylinder, which seems to represent the original pulp-cavity.

*Evolution.* They are frequent if not constant in embryo, and are arrested in growth at different periods of their evolution by the exertion of some recondite influence—probably according to Darwin's doctrine of natural selection. It is apparently conservative to state that a large share are arrested in growth during their fœtal life; others reach various stages of development after birth, while only a minority attain the period of eruption. The latter—the observable ones—alone demand attention, and may be classified according to the time of eruption into two classes:

1. Wolf-teeth of temporary dentition.
2. Wolf-teeth of permanent dentition.

This classification should not be mistaken to signify that temporary and permanent wolf-teeth exist, as they are never supplanted when once removed.

The first class are those very rudimentary teeth which appear between the tenth and twenty-fourth month. They seldom withstand the shedding of the first molar, or are so disturbed by this event as to render their removal quite certain at an early period.

The second class erupt between the second and fifth year, but may not pierce the gums for several years later, and in some instances remain covered with the gum through life.

According to location and size they may also be divided into two classes :

1. Those contiguous to the molar, usually large and well developed.

2. Those remote from the molar, usually small in size or rudimentary in structure.

The former are the best developed of all wolf-teeth. Whether they belong to the wolf-teeth of temporary dentition, and remain unmolested by eruption of the first molar, or erupt after this event has taken place, they always possess the distinctive features of mammalian teeth. They have firm alveolar walls, are surrounded by an alveolo-dental periosteum, and in many ways ably represent their predecessors. Their development seems to be greatly augmented by the influence of the first molar.

The latter (second class) are the most rudimentary of wolf-teeth, whether they appear during temporary or permanent dentition. They often remain covered by the gums, lose all dentinal qualities, and become incorporated with the maxilla. Even those whose fangs grow sufficiently to force the crown through the gums are deficient in structure and soon succumb to external influences.

Further research into their anatomical peculiarities, morphological characteristics, and their evolution tends to confirm the belief that they are retrogressive dentinal structures which will under suitable conditions become extinct.

Along these lines the effects of domestication, breeding, mode of life, and habits upon their size, frequency, and development should be noted. The wild horse, the broncho of the American plains, and the poorly-bred domestic horse have large, well-developed ones, possessing all the characteristics of typical mammalian teeth, while the finer breeds—the American trotter, the English hunter, the thoroughbred, and the larger breeds of draught-horse—have relatively small and rudimentary ones, often lacking a normal arrangement of the fundamental dental substances. In the former they are often actively concerned in mastication, while in the latter they usually succumb to external influences at an early age.

Confronted with this evidence, obtained by extensive observation, it would first appear that the doctrine of " natural selection " is not applicable to their retrogression, as all the influences which would naturally be expected to favor development, instead of degeneration, are exerted in the finer breeds; but when the variation in the quantity, quality, and texture of the food masticated by each is considered the soundness of the deduction becomes apparent.

Research as to their frequency in the different breeds is rather disappointing as compared with the other variations, yet they are somewhat more prevalent on the coarser breeds.

It is a matter of common observation that they are undergoing a "process of disappearance" in our modern well-bred horse, and are only retained upon the scene by the stubborn influence of heredity, and if the law of "natural selection" is applicable to their retrogression—and it no doubt is—its influence upon them is no less remarkable than the persistence with which they retain their identity after generations of disuse and uselessness to their possessors.

It cannot be disputed that there is a perceptible tendency toward enlargement of the diastema as breeds improve, and that the inferior maxilla—the seat of the bit—is no longer a common location for these teeth. It is, therefore, only reasonable to suppose that the "adaptive" influence of "natural selection" is instrumental in "clearing up" the interdental space of offensive agents.

*Their effect upon the eyes.* Among the laity the impression that wolf-teeth exert an evil influece upon the eyes is well established, while veterinarians hold somewhat varied opinions. The majority, however, are disinclined to entertain such views, or else dismiss the matter as unimportant. It must be admitted that if any relation exists between these organs and the visionary apparatus it is only remarkable for its remoteness. Yet the subject has been sufficiently agitated to warrant brief notice.

Along this line a number of theories have been advanced:

1. That the fang of the wolf-tooth infringes upon the supra-dental branch of the trigemini, and reflexly affects the organ of vision.

2. That "weeping-eyes" may be due to occlusion of the lachrymal conduit caused by eruption of wolf-teeth.

3. That irritations or catarrhal conditions incidental to their eruption affect the eyes by continuity of tissue.

It is readily seen that these deductions are of no value, however plausible they may seem from casual investigation. It would indeed require an elastic imagination to depict an extensive conjunctivitis caused by irritation of the dental nerve. The other two, in fact all these theories, are exploded by the fact that wolf-teeth erupt without any amount of irritation. They have never been observed to occasion any visible disturbance to the surrounding tissues. And, again, to argue that the circumscribed counter-irritation produced by their extraction could benefit an ocular disease is too speculative, if not entirely beyond reason.

The abortion of ocular phlegmasia following their extraction must be regarded as a mere coincident, or at least be viewed from an empirical standpoint until some substantial argument is advanced which will implicate them in the causation of such diseases.

*Treatment.* Being the rudiments of normal structures, never diseased, and, as far as observation goes, do not augment or cause disease of any character, it may seem inconsistent to prescribe surgical treatment for them. Yet for the following reasons their universal extraction should be recommmended, viz.:

1. To free the interdental space of offensive agents. Their location and unstable implantation render them susceptible to mechanical injury, and their crowns may cause abrasions of the buccal membrane. They thus interfere with mastication, and often are productive of disagreeable driving-habits, such as unilateral pulling, frothing, nervousness, etc. The necessity of obviating and palliating such habits cannot be over-estimated among highly bred, nervous animals, as their affability so largely depends upon a complacent acceptance of the bit.

2. To accede to the demands of an anxious client. And besides, during the treatment of ocular disease, the strategical veterinarian will never fail to recommend their extraction.

*Modus Operandi.* The operation of extracting wolf-teeth, seemingly too simple to warrant attention, is one of those many small matters by which a practitioner's gross ability is often measured, and from this standpoint alone should obtain more than passing notice from the circumspect veterinarian.

Like in the extraction of any tooth, the process requires the application of pressure, torsion, and outward force simultaneously and dexterously combined, and beside the operator must be mindful of the possible interference of the movable inferior maxilla. The amount of pressure and torsion must be measured by the stability of the tooth's substance, while the outward force must necessarily equal the firmness of its implantation.

In the case of the better developed ones, the operator assumes a position on the opposite side of the head. The hand containing the forceps is passed beneath the inferior maxilla to the side containing the tooth. In this position the tooth is grasped as near the neck as possible with the forceps forming a right angle with the long axis of the upper jaw. The instrument is held rigid to limit the outward motion of the lower jaw, then, with the proper application of pressure, torsion, and outward force, extraction is usually successful.

The rudimentary ones and those remote from the molars are best extracted by first disturbing the alveolar walls with a sharp separating-forceps before applying the regulation wolf-tooth instrument. Even in the case of well-developed ones the separating-forceps is valuable to render extraction certain. The tooth is loosened by forcing their edges between the molar and the wolf-tooth, then the regular forceps is applied, with success.

Fracture during extraction, while very common, is not usually serious from the practitioner's point of view, as the embedded part will soon become covered with the gum.

*Summary.* The following are a few deductions made from a study of these interesting structures, viz.:

1. That wolf-teeth represent the four anterior premolars (two superior and two inferior) of extinct genera, followed by an almost unbroken chain, from genera of the tertiary period through successive ages to the present horse.

2. That their retrogression is identical with that of the wisdom-teeth of man, which are undergoing the same process of degeneration in the more highly civilized races.

3. That they are retrogressing in the modern horse in accordance with the doctrine of natural selection.

4. That their retrogression is discernibly within the scope of modern observation.

5. That the theories that they are the result of accidental dentition (supernumerary), or of hyperdentinal development (supplementary), is not worthy of serious thought.

6. That they should be included in the category of normal structures, the same as the wisdom-tooth is included in the formula of human dentition.

7. That they are not etiological factors in diseases of the eyes, but may interfere with mastication and cause disagreeable driving-habits.

8. That they are less frequent and more rudimentary in the finer breeds.

9. That their development in size and structure depends upon their contiguity to the molar.

10. That their universal extraction should be practised by the veterinarian.

